# A Comprehensive Study on High-Temperature Oxidation Behavior of Ceramic Molds for Hot Embossing

**DOI:** 10.3390/ma15228045

**Published:** 2022-11-14

**Authors:** Youcheng Zhu, Feng Gong, Gao Yang

**Affiliations:** Shenzhen Key Laboratory of High Performance Nontraditional Manufacturing, College of Mechatronics and Control Engineering, Shenzhen University, Shenzhen 518060, China

**Keywords:** structural ceramics, high temperature, oxidation, molds, hot embossing

## Abstract

Structural ceramics are potential mold materials for hot embossing, due to their superior mechanical strength as well as low thermal expansion coefficient. However, the service time of molds, especially those in high-temperature hot embossing, strongly depends on their oxidation resistance. As a result, the oxidation behaviors of various ceramics (e.g., SiC, ZrO_2_, AlN, Al_2_O_3_, Si_3_N_4_ and WC) were investigated by conducting cyclic oxidation experiments in this study. Mass changes of ceramic samples thermal treated under different temperatures were measured by thermogravimeter (TGA) and precision electronic balance. The structural and chemical compositions of ceramic samples were detected by X-ray diffraction (XRD) and energy-dispersive X-ray spectroscopy (EDXS). The surface morphology of the samples was characterized by scanning electron microscopy (SEM), and the surface roughness of the samples was measured by white light interferometry. The mechanical properties of the samples were evaluated by a microhardness tester and nanoindentation instrument. It is noted that Al_2_O_3_ shows negligible oxidation within 1000 °C. ZrO_2_ maintains a decent surface roughness of below 32 nm and a stable hardness within 1000 °C. SiC has the highest hardness at high temperatures, and its surface roughness increases notably above 800 °C. The surface roughness of Si_3_N_4_ and AlN soars between 600 °C and 800 °C. The surface finish of WC is significantly deteriorated above 600 °C. Therefore, the appropriate embossing temperature of Al_2_O_3_ ceramics is below 1000 °C, that of ZrO_2_ ceramics is between 800 °C and 1000 °C, that of SiC ceramics is below 800 °C, that of Si_3_N_4_ and AlN ceramics is between 600 °C and 800 °C, and that of WC ceramics below 600 °C.

## 1. Introduction

Hot embossing [1] enables the fabrication of microstructures on various materials, such as polymers [2], metallic glasses [3], metals [4], and optical glasses [5], in a low-cost and efficient manner, which is widely used for the fabrication of microelectromechanical systems (MEMS) [6], microfluidics [7], and micro-optical components [8]. However, in hot embossing, molds are repeatedly subject to mechanical and thermal loads, so the selection of suitable mold materials becomes a critical issue. Compared to metals, ceramics exhibit superior mechanical strength [9,10], higher wear resistance [11], a relatively lower coefficient of thermal expansion [12], and thermal stability [13]. Therefore, they are regarded as promising mold materials for high-temperature hot embossing. However, the oxidation of ceramic molds at higher temperatures reduces their lifetime for hot embossing. Therefore, the oxidation behavior of ceramic materials is of great interest among scholars and engineers.

So far, WC ceramics have become a very promising precision molding material [14,15]. Adding Co, Ni and other dopants improves the bonding strength of the sintered WC ceramic, making it the most used ceramic mold material. However, dopants are readily oxidized at high temperatures, which causes damage to sintered WC ceramics. It is found that WC-Co ceramics degrade rapidly above 600 °C in the air environment [16]. Although binderless WC ceramics could be produced by making improvements in the sintering process [17], the oxidation of WC in a harsh high-temperature environment is still unavoidable.

Compared to WC ceramics, SiC, Si_3_N_4_, ZrO_2_, AlN, and Al_2_O_3_ ceramics have better high-temperature oxidation resistance. Narushima et al. [18] found that SiO_2_ would be generated on SiC and Si_3_N_4_ substrates at high temperatures. Furthermore, the generated SiO_2_ film filled the internal pores of the matrix so that the oxidation of the substrates was inhibited [19]. Hannink et al. [20] investigated the transformational toughening of ZrO_2_ ceramics, and observed a shift from the monoclinic to tetragonal structure of ZrO_2_ at 1000 °C, which led to an enhancement of the mechanical properties. Dutta et al. [21] studied the oxidation of AlN ceramics by using transmission electron microscopy and found that α-Al_2_O_3_ crystals were generated on the surface of sintered AlN ceramics at lower temperatures.

Many attempts have been made to quantify the oxidation behavior of ceramic materials. Ye et al. [22] used X-ray diffraction (XRD), energy dispersive X-ray spectroscopy (EDXS), and scanning electron microscopy (SEM) to analyze the phase, chemical composition, and surface morphology of high-entropy ceramics at high temperatures in air. Kane et al. [23] comprehensively discussed the oxidation kinetics, mechanisms and reaction products for borides, carbides, MAX-phases, and high-entropy ceramics. Miller-Oana and Corral [24] proposed a test method called dynamic nonequilibrium thermal gravimetric analysis (DNE-TGA) to study the effects of temperature, time, and gas flow on oxidation-induced mass change of ZrB2+SiC composites. This test method eliminates oxidation during the heating ramp. Wiame et al. [25] studied the thermal oxidation of zirconium nitride under oxygen by a combined study of XPS, DIFTS and TG-MS for a better understanding of the intermediate species encountered during the chemical reaction. Karlsdottir and Halloran [26] introduced a novel test method for characterization of ultra-high temperature ceramics in a rapid and low-cost manner. This method covers large temperature–time–composition parameters. Zhang et al. [27] systematically studied the oxidation mechanisms of Ta–Hf–C ternary ceramics at 1400–1600 °C in air by using densimeter, XRD, SEM, EDS and transmission electron microscopy (TEM).

In practice, the surface finish of hot embossed replica is largely determined by the surface roughness of the mold, and the wear resistance of the mold is closely related to its nano-hardness. However, the effects of oxidation on the surface roughness and nano-hardness of ceramic materials have been overlooked by previous studies. Moreover, a comprehensive comparison of oxidation behavior of various ceramic materials is lacking.

This study presents a comprehensive evaluation methodology for the oxidation behavior of structural ceramics by considering not only weight change, structures, chemical compositions, surface morphology, and reduced modulus, but also surface roughness and nano-hardness. Firstly, commonly used ceramic mold materials, such as SiC, ZrO_2_, AlN, Al_2_O_3_, Si_3_N_4_, and WC, were subjected to a 50-hour cyclic thermal treatment in air. After that, the mass changes of ceramic samples thermal-treated under different temperatures were measured by TGA and precision electronic balance. The crystalline phase and chemical compositions of ceramic samples were detected by XRD and EDXS, respectively. The surface morphologies of samples were comprehensively characterized by SEM), white light interferometry, and laser scanning confocal microscopy. The mechanical properties of samples were evaluated by nanoindentation, and microhardness tester. The research findings are expected to provide useful information for selection of appropriate mold materials for high-temperature hot embossing.

## 2. Materials and Methods

### 2.1. Materials

**Figure 1:**
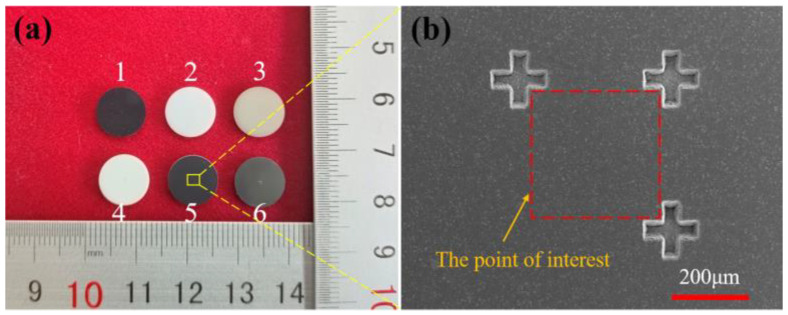
(**a**) Ceramic samples, 1–6: SiC, ZrO_2_, AlN, Al_2_O_3_, Si_3_N_4_, WC. (**b**) SEM image of point of interest that determined by cross marks.

As shown in Figure 1a, one-side polished SiC, ZrO_2_, AlN, Al_2_O_3_, Si_3_N_4_, WC ceramic discs that had a diameter of 10 mm and thickness of 1 mm were prepared by picosecond laser milling technology. In particular, the type of WC ceramic is YG8, with 92 wt% WC and 8 wt% Co. As shown in Figure 1b, three cross marks were generated on the surface of ceramic samples by picosecond laser milling as well. As a result, the rectangular area determined by these three marks is the point of interest for characterization in this study.

### 2.2. The Oxidation Experiments

Before the oxidation experiments, the ceramic samples and crucibles were subject to an ultrasonic bath for 20 min, and then dried at 100 °C for 30 min. After the cleaning treatment, the initial sizes and masses of the samples were measured by using a vernier caliper and an electronic balance respectively. It is noted that the accuracy of the vernier caliper and the electronic balance are 0.01 mm and 0.01 mg, respectively.

**Figure 2:**
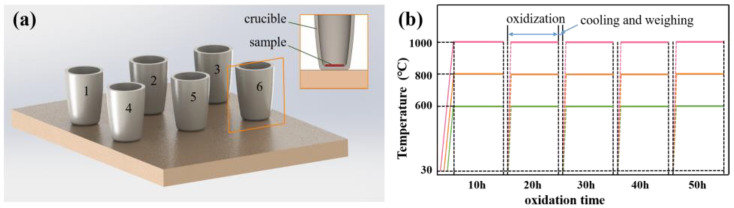
(**a**) The experimental layout, and (**b**) schematic diagram of heating histories.

Figure 2a illustrates the setup of oxidation experiments, where the ceramic samples were placed inside the crucible according to serial numbers, with their polished side upwards. In an oxidation experiment, six samples, one piece of each ceramic material, were placed in a muffle furnace so that experimental consistency was ensured. Since the embossing temperature of hot embossing is generally between 600 °C and 1000 °C, the temperatures of cyclic thermal treatments were set as 600 °C, 800 °C and 1000 °C. As shown in Figure 2b, the muffle furnace was heated from room temperature to oxidation temperature at a rate of 20 °C/min. After a soaking time of 10 h, the samples were taken out from the furnace and cooled to room temperature, followed by weight measurement using a precision electronic balance. After that, samples were again placed in the furnace for oxidation treatment. The above thermal treatment was repeated five times. As a result, the total time of cyclic thermal treatment was 5 h.

The oxidation resistance of ceramic samples was quantified by the weight change per unit surface area [23]:

(1) $$G=\frac{m_{2}-m_{1}}{S}$$

where *m*_1_ and *m*_2_ are the masses of the ceramic samples before and after the cyclic oxidation experiment, respectively, and *S* is the surface area of the ceramic samples.

### 2.3. Thermogravimetric Analysis

Most ceramic materials exhibit excellent oxidation resistance at temperatures below 800 °C, except for WC ceramics [12,16]. To investigate the oxidative stability of ceramic materials, the mass change of samples in air atmosphere was measured by using a thermogravimeter (TGA/DSC 3+, Mettler, Switzerland) that was heated from 30 °C to 800 °C at a rate of 10 °C/min.

### 2.4. Structural Analysis and Characterization of Oxidation Diffusion

To figure out the chemical reactions of ceramics at elevated temperatures in air, the surface of the ceramic samples before and after the oxidation experiment was scanned by the X-ray diffractometer (Mini-Flax 600, Rigaku, Japan) at a scanning speed of 4°/min and in the diffraction angle range of 10° to 90° for determining the phase change. However, the content of some oxides may have been too low to be detected by XRD. Therefore, SEM (Quanta FEG 450, FEI, San Jose, CA, USA) was used to observe the surface morphology of the ceramic samples in the marked area after high-temperature treatment and to discriminate the oxides based on the embedded EDXS system. The oxidation diffusion on the surfaces of ceramic samples at different temperatures was explored by observing the cross-section using SEM in the back-scattered electron mode.

### 2.5. Measurement of Surface Roughness

In hot embossing, the surface roughness of the preform is determined by that of the mold. As a result, the effects of oxidation treatment on the surface roughness of ceramic molds need to be evaluated. The surface roughness of most of the ceramic samples before and after oxidation experiments were measured by white light interferometry (Contour GT-X 3D, Bruker, Germany). Since WC ceramics undergo severe surface degradation at elevated temperatures, it was better to use a laser confocal microscope (VK-250, Keyence, Japan) to measure their surface roughness after the oxidation experiments. Furthermore, three measurements were taken for the point of interest that was determined by the three cross marks on the surfaces of all ceramic samples, and the average value of the surface roughness was calculated.

### 2.6. Test of Mechanical Properties

Mechanical properties of the material surface after high temperature treatment are also of importance for hot embossing. Hardness and elastic modulus affect the wear resistance and toughness, determining the service life of ceramic molds. Since ceramics are mostly hard and brittle materials, Vickers micro hardness tester (FM-ARS 9000, Future-Tech, Japan) and nanoindentation (TI-950, Hysitron, Eden Prairie, MN, USA) were used to measure the surface hardness and reduced modulus. The Vickers microhardness tester was used to measure the hardness of ceramic samples at 9 points in a 3 × 3 matrix with an interval of 0.5 mm by using 50 g load and holding for 5 s. The depth control mode of the nanoindentation instrument was used to measure the hardness and reduced modulus at 6 points in a 3 × 2 matrix with an interval of 50 μm. The indentation depth was set to be 2 μm. The measured data were averaged after removing the maximum and minimum values so that the measured error could be reduced.

## 3. Results and Discussion

### 3.1. Weight Changes

**Figure 3:**
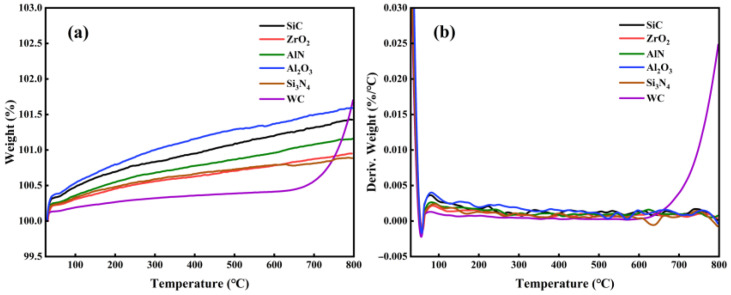
TGA results of structural ceramics below 800 °C. (**a**) weight change, and (**b**) derivative of weight.

**Figure 4:**
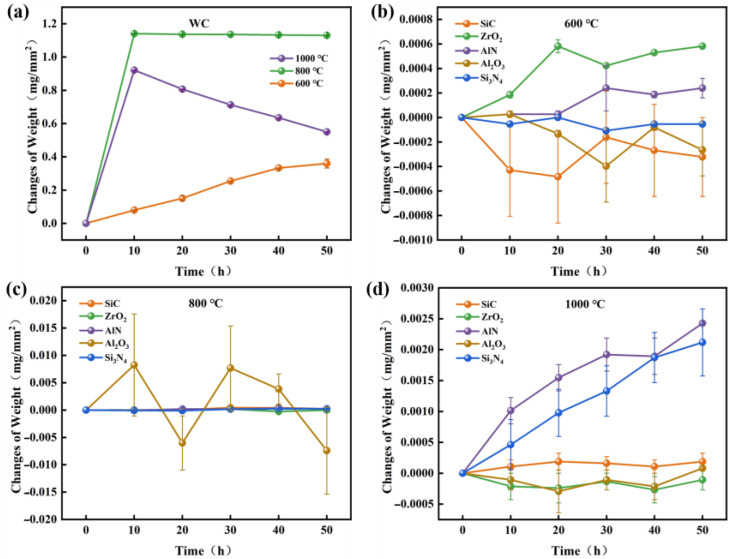
(**a**) Weight changes of WC at various oxidation temperatures. Weight change of various ceramics under oxidation temperatures of (**b**) 600 °C, (**c**) 800 °C, and (**d**) 1000 °C.

Figure 3 shows TGA results of various ceramics in air. It is noted that the oxidation of WC begins at 600 °C, while SiC, ZrO_2_, AlN, Si_3_N_4_, Al_2_O_3_ are barely oxidized below 800 °C. Figure 4 shows the weight changes of WC and other ceramics, which were measured by a precision electronic balance. At 600 °C, the weight of WC increased almost linearly with time. At 800 °C, the weight of WC reached its maximum value in 10 h and then remained stable. At 1000 °C, the weight of WC increased rapidly in the first 10 h, followed by a continues decrease. The decrease in the weight of WC at 1000 °C is attributed to the occurrence of oxidative weight loss. In agreement with the TGA results, weights of SiC, ZrO_2_, AlN, Al_2_O_3_ and Si_3_N_4_ ceramics barely changed at 600 °C and 800 °C, as seen in Figure 4b,c. When the oxidation temperature increased to 1000 °C, the weights of AlN and Si_3_N_4_ increased linearly with time, while the weights of SiC, ZrO_2_, and Al_2_O_3_ still showed little change. Therefore, SiC, ZrO_2_ and Al_2_O_3_ have the highest oxidation resistance, AlN and Si_3_N_4_ the next highest, and WC the lowest.

### 3.2. Structural Analysis

**Figure 5:**
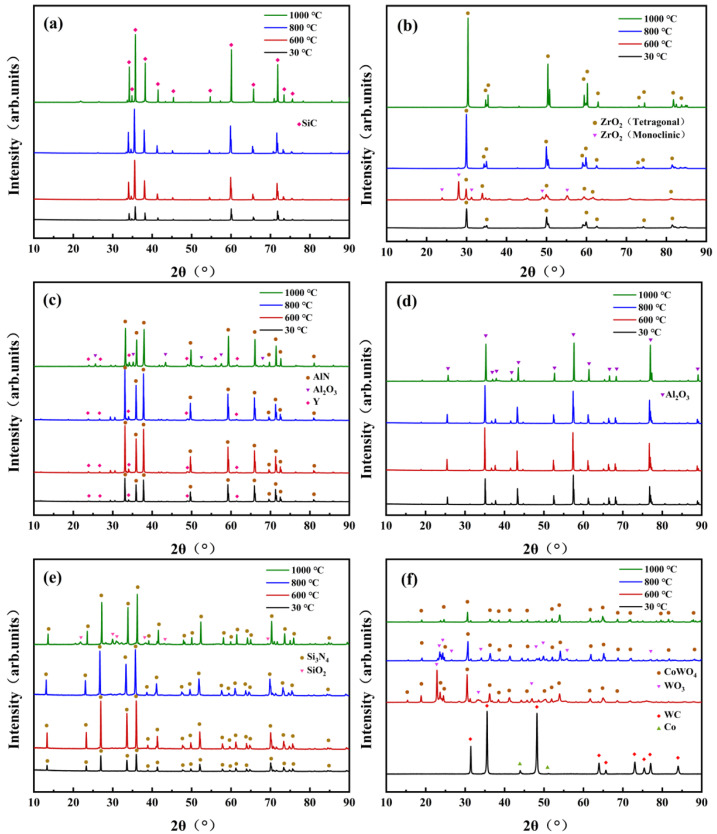
XRD patterns of ceramics before and after high-temperature treatment, (**a**) SiC, (**b**) ZrO_2_, (**c**) AlN, (**d**) Al_2_O_3_, (**e**) Si_3_N_4_, (**f**) WC.

Figure 5 shows the XRD pattern of various ceramic samples before and after oxidation experiments. As shown in Figure 5a,d, the diffraction peaks of SiC and Al_2_O_3_ ceramic samples after oxidation treatment had almost no change. Figure 5b shows that ZrO_2_ ceramic had only t-ZrO_2_ (tetragonal zirconia crystal) peaks at room temperature, and m-ZrO_2_ (monoclinic zirconia crystal) peaks occurred at 600 °C but disappeared at 800 °C and 1000 °C, which confirms the crystal phase transformation of ZrO_2_ ceramics at different temperatures. Figure 5c,e reveal that Al_2_O_3_ and SiO_2_ were generated on the surface of AlN and Si_3_N_4_ ceramic at 1000 °C, respectively. Figure 5f suggests that WC and Co dopants were oxidized to CoWO_4_ and WO_3_ at 600 °C. Furthermore, the content of WO_3_ decreased significantly as the temperature increased to 800 °C and eventually disappeared at 1000 °C.

### 3.3. Surface Morphology

**Figure 6:**
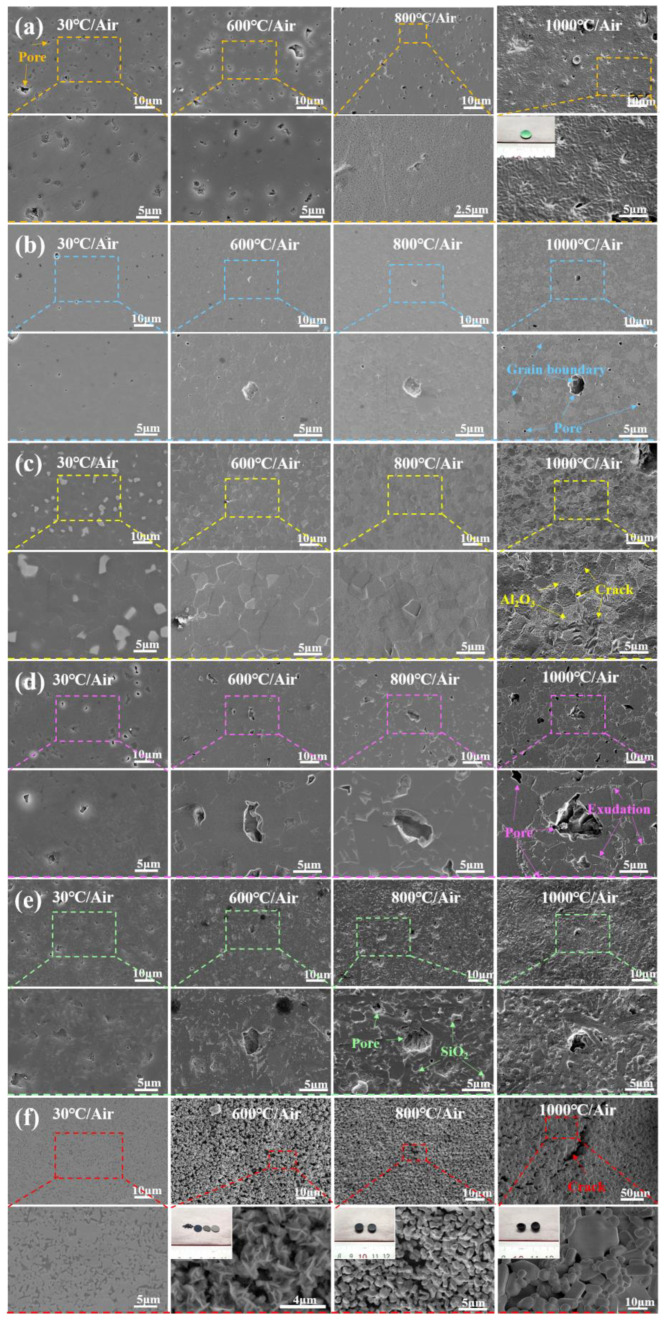
SEM images of surface morphology of ceramics after oxidation at 600 °C, 800 °C and 1000 °C, (**a**) SiC, (**b**) ZrO_2_, (**c**) AlN, (**d**) Al_2_O_3_, (**e**) Si_3_N_4_, (**f**) WC.

**Figure 7:**
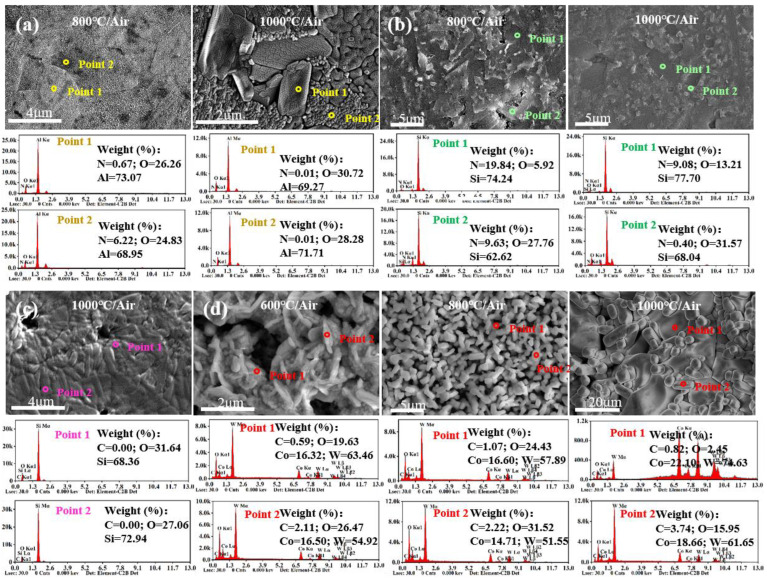
EDS analysis of elemental distribution on the surface of various ceramics: (**a**) AlN, (**b**) Si_3_N_4_, (**c**) SiC, (**d**) WC.

It is evident from Figure 6 that the surface morphology of different ceramics starts to significantly change at different temperatures due to the generation of oxides. Figure 6e and Figure 7b suggest that SiO_2_ was generated on the surface of Si_3_N_4_ ceramics at 600 °C. When the temperature reached 800 °C, more molten SiO_2_ appeared, which deteriorated the surface quality. At 1000 °C, the generated SiO_2_ sheets covered the surface of Si_3_N_4_ ceramics, reducing the number and diameter of pores. As shown in Figure 6f, the WC ceramic sample was split into three pieces by thermal stress impact at 600 °C, and a large number of pores were formed on the surface, which expedited the oxygen diffusion. At 800 °C, the WC ceramic was split into two halves, and the number of pores increased. The generated CoWO_4_ and WO_3_ were accumulated in granular and columnar forms. At 1000 °C, WC ceramic tended to shrink and became more brittle, causing the oxidation layer to fall off. After high-temperature oxidation treatment, Co elements were evenly distributed on the surface of the WC ceramic samples, while the C elements did not evenly exist on the surface, which resulted in the difference of microstructures, as shown in Figure 7d. When the temperature rose to 1000 °C, the number of grains and the content of O element of WC ceramics decreased, which confirmed the significant decrease in the content of WO_3_.

Compared with Si3N4 and WC ceramics, SiC and AlN ceramics underwent oxidation at higher temperatures. As shown in Figure 6a, the surface morphology of SiC ceramics was stable after 50 h of action under atmospheric atmosphere from 30 °C to 600 °C, but many pores appeared on the surface of SiC ceramic at 800 °C but were replaced by continuous oxide layers at 1000 °C, and diffraction spots appeared on the macroscopic surface at 1000 °C. The EDS spectra in Figure 7c show that the SiC ceramic oxide layer is composed of SiO_2_. As shown in Figure 6c and Figure 7a, many fine granular α-Al_2_O_3_ were generated on the surface of AlN ceramic at 800 °C. When it comes to 1000 °C, many fine cracks occurred on the surface of AlN ceramics that were covered by a large amount of Al_2_O_3_.

Since ZrO_2_ and Al_2_O_3_ are oxide ceramics, their surface morphologies were barely changed at below 800 °C. However, when the temperature reached 1000 °C, some tiny pores and clear grain boundaries appeared on the surface (see Figure 6b), and many precipitates along the grain boundaries were generated on the surface of Al_2_O_3_ ceramics (see Figure 6d).

### 3.4. Oxygen Diffusion Mechanism

**Figure 8:**
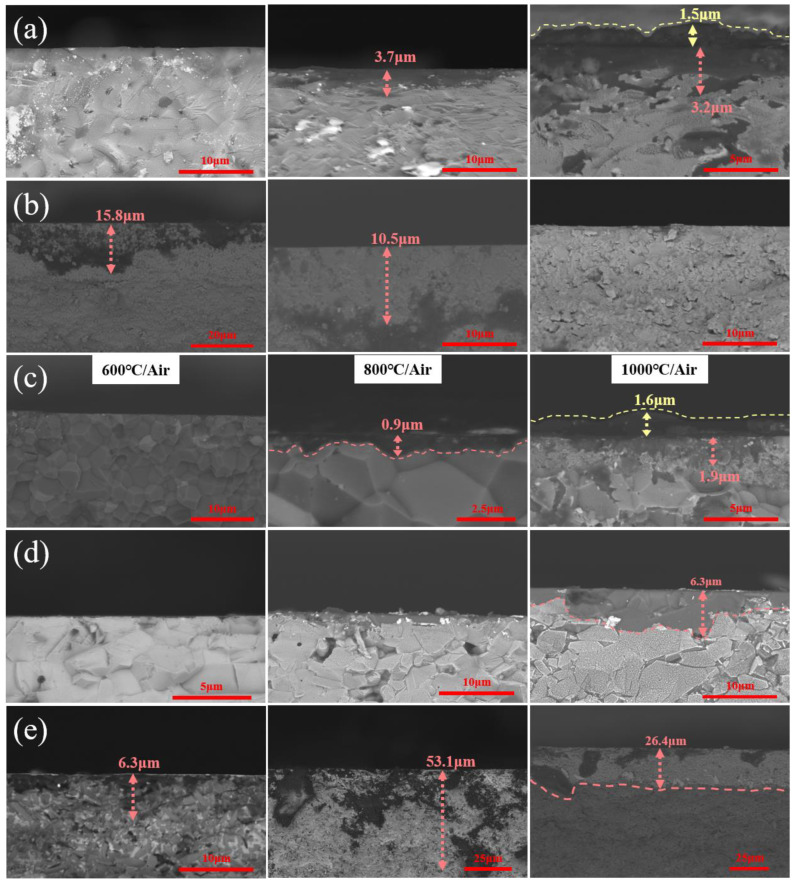
The oxidation diffusion of ceramic cross sections after oxidation, (**a**) SiC, (**b**) ZrO_2_, (**c**) AlN, (**d**) Al_2_O_3_, (**e**) Si_3_N_4_.

Figure 8 shows SEM images of the cross-section of ceramic samples after oxidation treatment. At modest temperatures, oxygen tends to diffuse into the matrix. As the temperature rises, oxides are generated on surfaces of nitride and carbide ceramic substrate due to the increase in surface energy and the presence of oxygen molecules. As a result, the oxidation depth of SiC and AlN ceramics increased steadily with the increase in oxidation temperature, as seen in Figure 8a,c. The surface of Si_3_N_4_ ceramics began to oxidize at 600 °C and form a uniform oxidation layer, having a relatively large thickness of 26.4 μm at 1000 °C (see Figure 8e). In contrast, the depth of m-ZrO_2_ generated on the ZrO_2_ ceramic substrate was 15.8 μm at 600 °C and 10.5 μm at 800 °C, and it was completely transformed into t-ZrO_2_ at 1000 °C (see Figure 8b). Since Al_2_O_3_ ceramic itself is an oxide, hardly reacting with air, there was no oxidized sediment on the surface, as shown in Figure 8d.

### 3.5. Surface Roughness

**Figure 9:**
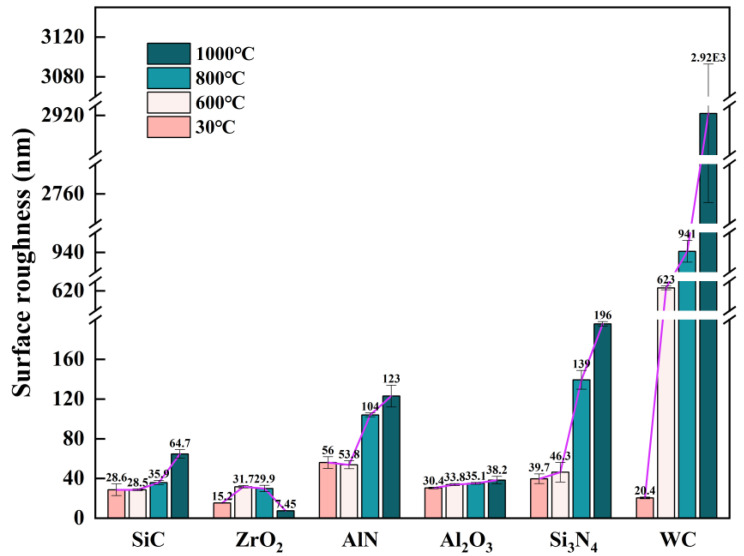
Surface roughness of ceramics after the action of 600 °C, 800 °C and 1000 °C, (**a**) SiC, (**b**) ZrO_2_, (**c**) AlN, (**d**) Al_2_O_3_, (**e**) Si_3_N_4_, (**f**) WC.

Figure 9 compares the surface roughness of various ceramics before and after oxidation treatment. It is noted that the surface roughness of all ceramic samples before oxidation was in the range of 15–60 nm. Owing to the extraordinarily excellent oxidation resistance, the surface roughness of Al_2_O_3_ ceramics hardly changed within 1000 °C. The surface roughness of SiC remained stable below 800 °C, but doubled at 1000 °C. AlN and Si_3_N_4_ ceramics had relatively stable surface roughness below 600 °C. However, their surface roughness exceeded 100 nm when the oxidation temperature was above 800 °C, which is not suitable for hot embossing optical surfaces. WC had the worst oxidation resistance among these ceramics. As a result, its surface roughness increased significantly at high temperatures: 623 nm at 600 °C and 941 nm at 800 °C. Moreover, the WC samples oxidized at 1000 °C were broken into small pieces. Apparently, as the oxidation temperature rises, the surface roughness of most ceramics increases, except for the ZrO_2_ ceramic. The surface roughness of ZrO_2_ ceramics rises sharply to 31.72 nm at 600 °C, but decreases slightly to 29.9 nm at 800 °C, and eventually drops to 7.45 nm at 1000 °C. It is speculated that the increase in surface roughness at 600 °C is caused by the precipitation of m-ZrO_2_ on the surface of ZrO_2_ ceramics, while the decrease in surface roughness at higher temperatures is resulted from transition of m-ZrO_2_ to t-ZrO_2_.

### 3.6. Mechanical Properties

**Figure 10:**
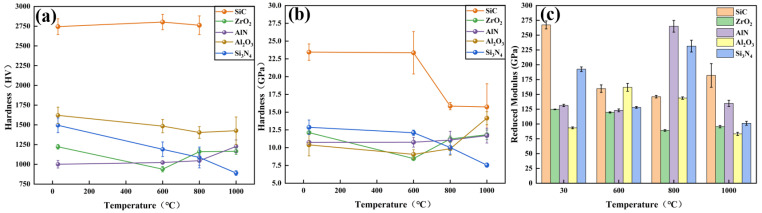
(**a**) Microhardness measured by Vickers hardness tester, (**b**) hardness and (**c**) reduced modulus measured by nanoindentation instrument.

Figure 10a shows the Vickers hardness of ceramic samples that were thermally treated at various temperatures. It is noted that SiC had the highest hardness and remained stable from room temperature to 800 °C. However, the SiC sample oxidized at 1000 °C was so brittle that the measurement of Vickers hardness failed. The hardness of Si_3_N_4_ ceramics continued to decrease from ~1500 HV to ~900 HV with the increase in temperature, which is attributed to the SiO_2_ generated on the surface of the Si_3_N_4_ substrates. The hardness of Al_2_O_3_ continued to drop from 600 °C to 800 °C, but rose slightly at 1000 °C. At room temperature, the Vickers hardness of AlN was lower than that of Al_2_O_3_. At elevated temperatures, the Al_2_O_3_ generated on the surface of AlN increased the microhardness. As a result, the Vickers hardness of AlN ceramics rose as the temperature increased. The hardness of ZrO_2_ ceramics decreased remarkably from room temperature to 600 °C but increased steadily from 600 °C to 800 °C, and then stabilized after 800 °C. Based on the XRD analysis, the generation of m-ZrO_2_ crystals on ZrO_2_ substrate reduced the surface hardness. Furthermore, the surface roughness of WC after oxidation treatment was quite high, leading to the failure of the microhardness measurement. As a result, the microhardness data of WC are not provided.

Figure 10b shows the hardness and reduced modulus of various ceramics, which were measured by the nanoindentation instrument. It was found that the trend of curves of ZrO_2_, AlN, Al_2_O_3_ and Si_3_N_4_ is similar to those measured by the Vickers hardness tester. However, the hardness of SiC ceramics dropped abruptly at 800 °C, which differs from that measured by the Vickers hardness tester. This difference is attributed to the different measurement modes and detection depths of the nanoindentation instrument and Vicker hardness tester.

The reduced modulus of SiC and Al_2_O_3_ ceramics shows opposite tendencies with the increase in temperature, as seen in Figure 10c. SiC had the highest reduced modulus at room temperature and 1000 °C, while Al_2_O_3_ showed the highest reduced modulus at 600 °C. Interestingly, the reduced modulus of silicon-based ceramics (i.e., SiC and Si_3_N_4_) decreased significantly at 600 °C, whereas the reduced modulus of nitride ceramics (i.e., AlN and Si_3_N_4_) showed a rapid increase at 800 °C. It is assumed that the change in reduced modulus might be driven by the Si and N elements in the ceramics.

## 4. Conclusions

In this study, the oxidation behaviors of SiC, ZrO_2_, AlN, Al_2_O_3_, Si_3_N_4_ and WC ceramics were investigated by cyclic oxidation tests at 600 °C, 800 °C and 1000 °C. By characterizing the weight change, crystalline phase, chemical composition, surface morphology, surface roughness and mechanical properties of ceramics before and after high-temperature oxidation treatment, some conclusions were drawn as follows

- WC has the most significant weight change at high temperatures. At 1000 °C, the weights of AlN and Si_3_N_4_ start to significantly increase, while the weight changes of SiC, ZrO_2_ and Al_2_O_3_ ceramics are still imperceptible.
- Si_3_N_4_ and WC ceramics are oxidized at 600 °C, while SiC and AlN ceramics start to oxidize at 800 °C. ZrO_2_ ceramic shows phase transform into m-ZrO_2_ at 600 °C, and Al_2_O_3_ ceramic generates precipitate at 1000 °C.
- At modest temperatures, oxygen tends to diffuse into the matrix of ceramics. As the temperature rises, oxides are generated on surfaces of nitride and carbide ceramic substrate due to the increase of surface energy and the presence of oxygen molecules.
- The surface roughness of Al_2_O_3_ ceramics hardly changes within 1000 °C. The surface roughness of SiC remains stable below 800 °C, but doubles at 1000 °C. The surface roughness of AlN and Si3N4 ceramics are relatively stable below 600 °C, but exceed 100 nm when the oxidation temperature is above 800 °C.
- The surface roughness of ZrO_2_ ceramic rises sharply to 31.72 nm at 600 °C, due to the precipitation of m-ZrO_2_ on the surface of ZrO_2_ ceramic. However, the surface roughness decreases slightly to 29.9 nm at 800 °C, and eventually drops to 7.45 nm at 1000 °C, which is resulted from the transition of m-ZrO_2_ to t-ZrO_2_.
- SiC ceramics have the highest hardness and remain stable at elevated temperatures. As the temperature increases, oxidized Si_3_N_4_ ceramics have lower hardness, while oxidized AlN ceramics have the opposite trend. The hardness of Al_2_O_3_ continues to drop from 600 °C to 800 °C, but rises slightly at 1000 °C.

Since the oxidation of ceramic molds limits their service life for hot embossing, the working temperature of Al_2_O_3_ ceramics is below 1000 °C, that of ZrO_2_ ceramics is between 800 °C and 1000 °C, that of SiC ceramics is below 800 °C, that of Si_3_N_4_ and AlN ceramics is between 600 °C and 800 °C, and that of WC ceramics is below 600 °C. In future work, the structures and chemical compositions of oxidized ceramics will be further determined by TEM and XPS to reach more reliable conclusions.
